# Bay breeze influence on surface ozone at Edgewood, MD during July 2011

**DOI:** 10.1007/s10874-012-9241-6

**Published:** 2012-11-16

**Authors:** Ryan M. Stauffer, Anne M. Thompson, Douglas K. Martins, Richard D. Clark, Daniel L. Goldberg, Christopher P. Loughner, Ruben Delgado, Russell R. Dickerson, Jeffrey W. Stehr, Maria A. Tzortziou

**Affiliations:** 1 Department of Meteorology, The Pennsylvania State University, University Park, PA 16802 USA; 2 Department of Earth Sciences, Millersville University, Millersville, PA 17551 USA; 3 Department of Atmospheric and Ocean Science, University of Maryland, College Park, MD 20742 USA; 4 Earth System Science Interdisciplinary Center, University of Maryland, College Park, MD 20742 USA; 5 NASA Goddard Space Flight Center, Greenbelt, MD 20771 USA; 6 Joint Center of Earth Systems Technology, University of Maryland-Baltimore County, Baltimore, MD 21250 USA

**Keywords:** Ozone, Bay Breeze, Pollution, Edgewood, Mid-Atlantic, DISCOVER-AQ

## Abstract

Surface ozone (O_3_) was analyzed to investigate the role of the bay breeze on air quality at two locations in Edgewood, Maryland (lat: 39.4°, lon: −76.3°) for the month of July 2011. Measurements were taken as part of the first year of NASA’s “Deriving Information on Surface Conditions from Column and Vertically Resolved Observations Relevant to Air Quality” (DISCOVER-AQ) Earth Venture campaign and as part of NASA’s Geostationary for Coastal and Air Pollution Events Chesapeake Bay Oceanographic campaign with DISCOVER-AQ (Geo-CAPE CBODAQ). Geo-CAPE CBODAQ complements DISCOVER-AQ by providing ship-based observations over the Chesapeake Bay. A major goal of DISCOVER-AQ is determining the relative roles of sources, photochemistry and local meteorology during air quality events in the Mid-Atlantic region of the U.S. Surface characteristics, transport and vertical structures of O_3_ during bay breezes were identified using in-situ surface, balloon and aircraft data, along with remote sensing equipment. Localized late day peaks in O_3_ were observed during bay breeze days, maximizing an average of 3 h later compared to days without bay breezes. Of the 10 days of July 2011 that violated the U.S. Environmental Protection Agency (EPA) 8 h O_3_ standard of 75 parts per billion by volume (ppbv) at Edgewood, eight exhibited evidence of a bay breeze circulation. The results indicate that while bay breezes and the processes associated with them are not necessary to cause exceedances in this area, bay breezes exacerbate poor air quality that sustains into the late evening hours at Edgewood. The vertical and horizontal distributions of O_3_ from the coastal Edgewood area to the bay also show large gradients that are often determined by boundary layer stability. Thus, developing air quality models that can sufficiently resolve these dynamics and associated chemistry, along with more consistent monitoring of O_3_ and meteorology on and along the complex coastline of Chesapeake Bay must be a high priority.

## Introduction

### Surface ozone regulation

Surface ozone (O_3_) is a secondary photochemical pollutant formed from a number of reactions involving volatile organic compounds (VOCs), nitrogen oxides (NO_x_ ≡ NO + NO_2_) and sunlight. Ozone production and surface mixing ratios are also dependent on a complex combination of meteorological processes including incoming solar radiation, temperature, humidity, boundary layer height, and surface wind speed (Comrie 1990; Sillman and Samson 1995; Bloomer et al. 2009; Steiner et al. 2010; Banta et al. 2011). The ability of O_3_ to negatively affect respiratory systems in humans and vegetation photosynthesis (Krupa and Manning 1988; Burnett et al. 1994; Jerrett et al. 2009) has led the U.S. Environmental Protection Agency (EPA) to adopt O_3_ as a criteria pollutant. Ozone is regulated by the current standard of 75 parts per billion by volume (ppbv), calculated using the daily maximum of an 8 h running mean (National Ambient Air Quality Standards or NAAQS, EPA).

### Bay breeze association with coastal air quality

Coastal areas near urban centers are often subjected to poor air quality through either direct downwind transport of pollutants (Angevine et al. 2004), in-situ production of O_3_, or a recirculation brought about by a bay or sea breeze (Banta et al. 2005). Bay or sea breeze (bay breeze from hereon) circulations and their association with poor air quality have been extensively studied in various locations throughout the world (Gangoiti et al. 2002; Darby 2005; Darby et al. 2007; Zhang et al. 2007; Rappenglück et al. 2008; Wu et al. 2010; Banta et al. 2011; Loughner et al. 2011; Martins et al. 2012). This study will focus on the impact of bay breezes on air quality in the northern Chesapeake Bay region in the Mid-Atlantic United States.

The meteorological conditions that are typically associated with the formation of bay breeze circulations also favor enhanced O_3_ production. Warm temperatures and strong sunlight accelerate the reactions that produce O_3_, and the calm or light winds necessary to allow the bay breeze to become dominant can lead to additional accumulation of O_3_ through reduced boundary layer venting and dispersion. Differential heating of the land and water leads to hydrostatic pressure gradients that force the movement of near-surface air from water to land during the day under relatively quiescent large-scale flow (Miller et al. 2003). As the land cools much faster than water at night, a reversal of the temperature and pressure gradients can cause flow in directions opposite the afternoon bay breeze. This aids in the early morning transport of emissions from over the land to over the bay waters. The O_3_ produced from these emissions is then recirculated back over coastal sites via the bay breeze (Wang et al. 2001; Ding et al. 2004).

In addition to comparable meteorological conditions favoring both bay breeze initiation and O_3_ production, differences in the air masses and behavior of O_3_ between land and water surfaces introduce further effects. Mixing depths over water are typically lower than those found over land during the daytime due to water having a much higher heat capacity compared to land (Hsu and Blanchard 2003). When the stable over-water air mass is advected onto land, the mixing height is typically determined by the height of the Thermal Internal Boundary Layer when formed (TIBL; Raynor et al. 1979). TIBLs form when the stable air mass modified by its passage over a water body contacts the land surface and forms separate layers of stability within the terrestrial boundary layer. TIBLs are frequently much shallower than the terrestrial boundary layer. Through reduced mixing, this shallow, more statically stable zone concentrates pollutants that continue to be emitted from the terrestrial surfaces. Mixing depths in coastal areas of the Mid-Atlantic and Chesapeake Bay region have been shown to decrease significantly from inland sites (Berman et al. 1999), often attributed to bay or sea breeze effects. The pollutants concentrated in these shallow, stable layers are then trapped and transported inland via the bay breeze (Banta et al. 2005).

Further aiding the build-up of O_3,_ characteristic deposition velocities of O_3_ over water are six times smaller than those over land (~0.07 cm s^−1^ vs. ~0.4 cm s^−1^ respectively; Lenschow et al. 1981; Lenschow et al. 1982; Hauglustaine et al. 1994; Wesely and Hicks 2000), yielding minimal O_3_ loss onto a water surface compared to a land surface through deposition. Compounding these factors with ideal meteorological conditions leads to the accumulation of high O_3_ mixing ratios and advection to nearby coastal locations.

This study uses numerous instruments from the July 2011 DISCOVER-AQ and Geo-CAPE CBODAQ deployments to investigate the bay breeze phenomenon. The goals are to examine the spatial and temporal evolution of O_3_ as it is affected by the bay breeze, as well as the vertical structure of O_3_ and atmospheric stability during these events.

## Methods

Table 1 summarizes the measurements utilized in this study.

**Table 1:** Measurements utilized in this study with uncertainties/accuracies and the platforms on which they were deployed

Instrument, Model	Platform	Measurement	Uncertainty/Accuracy
TECO Inc., 49C	NATIVE	Surface O_3_	±2 %
TECO Inc., 42C-Y	NATIVE	NO/NO_y_	±3 %
RM Young, #05103	NATIVE	Wind Speed, Direction	±0.3 m/s, ±3°
RM Young, #41382	MDE Trailer	Temperature, RH	±0.3 °C, ±1 % (at 23 °C)
ENSCI Corp., Model 2Z	Free Balloon	Ozone	<10 %
Intermet, iMet-1	Free Balloon	Temperature, RH, Pressure	±0.3 °C, ±5 %, ±1.8 hPa (400–1070 hPa), ±0.5 hPa (4–400 hPa)
Sigma Space, Mini-MPL	NATIVE	Backscattered 532 nm	±2 % for all altitudes
Teledyne, 400A	MABL	Surface O_3_	±2 %
ScinTec, windRASS	MABL	Vertically Resolved Wind Speed, Direction	0.3–0.5 m/s, <1.5° (at <2.0 m/s)
Vaisala, TTS111	Tether Balloon	Temperature, RH, Pressure	±0.5 °C, ±5 %, ±1.5 hPa
2B-Technologies, 205	Tether Balloon	Ozone	±2 %
NCAR 4 Channel Chemiluminescence	P3-B	Ozone	±5 %
General Eastern, 1011B	P3-B	Temperature	±0.2 °C
Rosemount, 102	P3-B	Dew/Frost Point	±0.6 °C
Rosemount, MADT 2014	P3-B	Pressure	±0.25 hPa
TECO Inc., 49C	Cessna 402B	Ozone	±1.0 ppbv (estimated)
Vaisala, PTU 300	Cessna 402B	Temperature, RH, Pressure	±0.2 °C, ±1 %, ±0.2 hPa (at 20 °C)
TECO Inc., 49	NOAA SRVx	Surface O_3_	±5 % (estimated)

### Edgewood, Maryland

The O_3_ design value, defined as the 3-year average of the fourth highest annual 8-h maximum O_3_ mixing ratio, determines compliance with the EPA NAAQS. In the Baltimore Non-Attainment Area (NAA; Maryland Department of the Environment), the highest O_3_ design value is consistently observed in Edgewood. In fact, Edgewood is the only monitor within its EPA Ozone Transport Region (OTR), an area stretching from Virginia to Massachusetts, that currently breaches the previous NAAQS of 84 ppbv enacted in 1997. Edgewood is also often the lone monitor in the Baltimore NAA to exceed the 75 ppbv standard on particular days (Landry 2011). It is believed that the bay breeze plays a major role in the exceptionally poor air quality at Edgewood (Piety 2007).

**Fig. 1:**
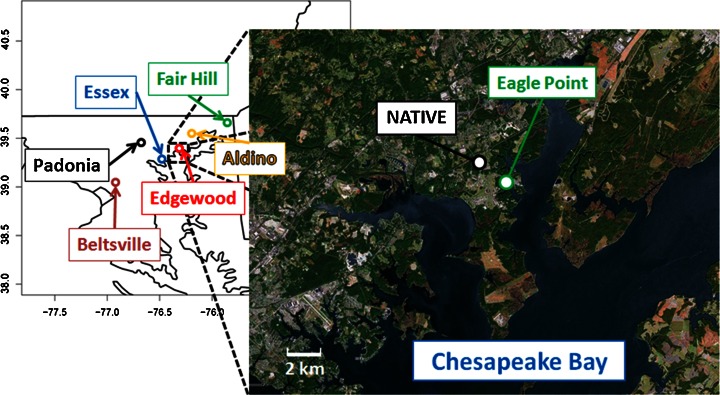
Map of the DISCOVER-AQ ground sites used in this study. All sites are located in Maryland. Edgewood area is expanded to show relative locations of NATIVE and Eagle Point to the Chesapeake Bay

Air quality measurements for DISCOVER-AQ (http://www.nasa.gov/discover-aq) 2011 were collected during the month of July at several pre-existing Maryland Department of the Environment (MDE) locations in the Baltimore-Washington Metropolitan Area (Fig. 1). Ship measurements of air quality in the upper Chesapeake Bay were collected during 11–20 July 2011 as part of NASA’s oceanographic campaign Geo-CAPE CBODAQ (http://neptune.gsfc.nasa.gov/osb/index.php?section=250). Much of the results presented in this manuscript are from measurements collected at the Edgewood side of the Aberdeen Proving Ground (APG), in Aberdeen and Edgewood. APG is a U.S. Army facility with a population of just over 3,000 on site. Aberdeen and Edgewood proper have populations of approximately 15,000 and 25,000 respectively, but are often influenced chemically by transport of NO_x_ and VOC emissions from the greater Baltimore-Washington Metropolitan Area, a Combined Statistical Area, or grouping of metropolitan areas, with 9 million residents. It is noted that the recent transfer of 50,000 people or more due to the Defense Base Realignment and Closure (BRAC) program is expected to add to the local population (Fry, http://articles.centermaryland.org). Thus, this study provides important baseline air quality information for the Edgewood area.

### Edgewood mobile trailer measurements

The Nittany Atmospheric Trailer and Integrated Validation Experiment (NATIVE; lat: 39.410°, lon: −76.297°; see Martins et al. 2012 for full description), a mobile platform designed for atmospheric process studies and comparison of satellite, air quality and ground-based measurements, provided 1 min averaged in-situ chemical measurements at Edgewood, including O_3_, NO/NO_2_/NO_x_, total reactive nitrogen (NO_y_), sulfur dioxide (SO_2_) and carbon monoxide (CO). Instruments for measuring atmospheric pressure, wind speed/direction, NO_2_ photolysis rates and UV radiation were also part of the NATIVE payload. Temperature and relative humidity were measured on a MDE trailer (FIPS Code: 240251001) located approximately 10 m from NATIVE.

Free-flying ozonesondes were also launched from the NATIVE site at Edgewood. The ozonesondes used the Electrochemical Concentration Cell (ECC; Komhyr 1969) technique. Ozonesondes were launched with an attached radiosonde (International Met Systems; iMet-1) equipped with temperature, humidity and pressure sensors. Ozonesondes were either launched twice a day to correspond with NASA P3-B aircraft spirals on flight days, or once daily to coincide with the Aura satellite overpass at approximately 1330 Eastern Standard Time (EST; all times presented are in EST due to the photochemical nature of O_3_ production).

Additional instruments were added to the NATIVE platform for the 2011 DISCOVER-AQ campaign. Sigma Space Mini Micropulse Lidar (MiniMPL) provided elastic lidar observations at Edgewood. The MiniMPL lidar transmitter consists of a 532 nm (frequency-doubled Nd: YAG laser) that operates at a 5,000 Hz repetition rate and average pulse energy of 3–4 μJ. The receiver consists of an 80 mm telescope that collects co-polarized backscattered light. The output from the telescope is conveyed to a photon counting silicon avalanche photo-diode (APD) manufactured by Perkin-Elmer. The raw data is converted to aerosol attenuated backscatter by taking into account instrumental factors that include corrections for detector dead time, geometrical overlap, background subtraction, and range-squared normalization. Recorded lidar profiles have temporal and vertical resolution of 1 min and 30 m, respectively.

The MPL lidar is a powerful tool for visualizing, in real time, with high temporal and spatial resolution, the planetary boundary layer (PBL). The PBL contains greater aerosol concentration than the above free troposphere because the aerosols, mainly produced near the surface, are trapped by a potential temperature inversion. The backscatter signal strength is dramatically reduced when returning from the free troposphere. A covariance wavelet technique (CWT) was applied to the lidar signal to determine the height of these gradients in the backscatter profiles. These heights were used as a proxy for the height of the PBL (Davis et al. 2000; Brooks 2003).

### Eagle Point Edgewood measurements

The Millersville University Atmospheric Boundary Layer (MABL) facility was deployed to Eagle Point, APG approximately 2.7 km SE of NATIVE (Fig. 1; lat: 39.396°, lon: −76.269°) to provide surface and boundary layer observations in support of DISCOVER-AQ. MABL is an integrated multi-platform facility that includes surface trace gas analyzers and particle scattering instrumentation, a 4 m tower for measuring surface fluxes, MPL, and an acoustic sodar with radio acoustic sounding extension. These surface instruments integrate with aloft measurements obtained using a tethered aerostat, rigged to carry meteorological and air quality monitoring instrumentation. Trace gas concentrations were measured using a suite of Teledyne API analyzers; O_3_ Model 400A, NO/NO_2_/NO_X_ Model 200A, SO_2_ Model 100A, and CO Model 300A, and sampled from the manifold at their respective specified volumetric flow rates at atmospheric pressure. Methods are identical to those of NATIVE for these gases. The response and concentration of each instrument was monitored at least weekly using the NATIVE multigas calibration system. A ScinTec Acoustic Sodar windRASS was used to produce time-height series of u, v, w wind components and virtual temperature. Self-tests were conducted daily on the sodar to assure within-spec operation. The tethered aerostat was used to carry aloft meteorological sensors, which measured temperature, pressure, relative humidity, wind speed and wind direction using a Vaisala TTS111 tethersonde. In addition, the aerostat deployed 2B-Technologies Inc. trace gas analyzers for profiles of O_3_, NO, and NO_x_. Daily comparisons were made with surface analyzers. The 2Btech Model 205 measures O_3_ using UV absorption at 254 nm every 2 s and the measurements were averaged to 5 min to conform to the NO/NO_X_ sampling rate. The Model 205 has a baseline drift of <1 ppbv day^−1^; sensitivity drift of <1 % day^−1^, and resolution of 0.1 ppbv. Uncertainty in O_3_ was 2 %. NO was obtained with a 2Btech Model 410 and is based on a selective reaction with O_3_ and the resulting change in UV absorption from the O_3_ depletion. A Model 401 Molybdenum converter was used to obtain NO_X_. Data were averaged to 5 min with uncertainty of 2 %.

### MDE O_3_ measurements

Additional O_3_ measurements were taken at existing MDE locations within the DISCOVER-AQ domain at Aldino (FIPS Code: 240259001), Beltsville (240330030), Essex (240053001), Fair Hill (240150003), and Padonia (240051007), MD. In addition to Edgewood, these sites were the locations where NASA’s P3-B collected vertical profile spirals during the campaign. Aldino, Beltsville, and Essex are equipped with Thermo Scientific 49i or 49C O_3_ analyzers and Fair Hill and Padonia used Ecotech 9810 O_3_ analyzers. All surface O_3_ instruments in this study employ a UV-Photometry method where mixing ratios are determined from UV extinction through a sample cell.

### Aircraft measurements

NASA’s P3-B and the University of Maryland’s Cessna 402B aircraft were deployed during the DISCOVER-AQ campaign to obtain vertical profiles of air quality measurements over ground sites. The aircraft occasionally performed profile spirals and transects at low altitudes across the Chesapeake Bay. O_3_ measurements from the P3-B were made using the National Center for Atmospheric Research (NCAR) 4-Channel Chemiluminescence Instrument, where ambient O_3_ is reacted with excess NO to produce photons. The photons are then counted using a cooled photomultiplier tube (PMT) and converted into an O_3_ mixing ratio in 1 s averages with 5 % uncertainty. The Cessna 402B employed a UV-Photometry method using a Thermo Scientific 49C O_3_ analyzer to produce 10 s averaged O_3_ mixing ratios with an estimated 1 ppbv uncertainty.

### Ship measurements

The NOAA SRVx National Marine Sanctuary Test and Evaluation Vessel sailed the Chesapeake Bay from 11 to 20 July as part of the Geo-CAPE CBODAQ field campaign. Air and water quality measurements were made onboard the ship, including in-situ O_3_ observations over the water surface. Ozone on SRVx was measured using UV-Photometry with a Thermo Scientific 49 O_3_ analyzer averaged to 10 s with an estimated 5 % uncertainty.

### Bay breeze identification

Meteorological criteria were set specifically for the Edgewood site and evaluated for each day to determine if a bay breeze circulation occurred. These requirements were: (1) a daytime wind shift from calm or offshore (southwesterly to easterly directions, moving clockwise from ~230° to 090°) to onshore (easterly to southwesterly directions, moving clockwise from ~100°–220°), (2) an increase in dew point temperature of at least 1 °C within 1 h after onset of wind shift, and (3) the lack of a meso-or synoptic scale front analyzed by the Hydrometeorological Prediction Center (HPC; hpc.noaa.gov). A criterion involving a steadying or decreasing dry bulb temperature was considered as in Sikora et al. (2010), but failed to discriminate further between bay breeze days and non-bay breeze days, and was thus eliminated.

## Results

### Bay breeze days

Bay breezes were identified on 02, 05, 23, 26 and 29 July. Four additional days (07, 19, 22, and 31 July) exhibited evidence of bay breeze initiation that was then inhibited by clean mid-tropospheric air from a local thunderstorm gust front, or the thunderstorm itself. Such days are classified separately as “interrupted” days. An overview of surface O_3_ during the entire month of July 2011 at Edgewood is provided in Table 2. Color codes based on the EPA National Ambient Air Quality Standard (NAAQS) are presented. There were a total of ten exceedances (Code Orange or higher) of the EPA 8-h O_3_ standard of 75 ppbv at Edgewood during July 2011. Of these ten exceedances, eight occurred on either bay breeze days or “interrupted” days, with the only two Code Red days (defined as 96 to 115 ppbv 8-h average O_3_) on 02 and 22 July.

**Table 2:**
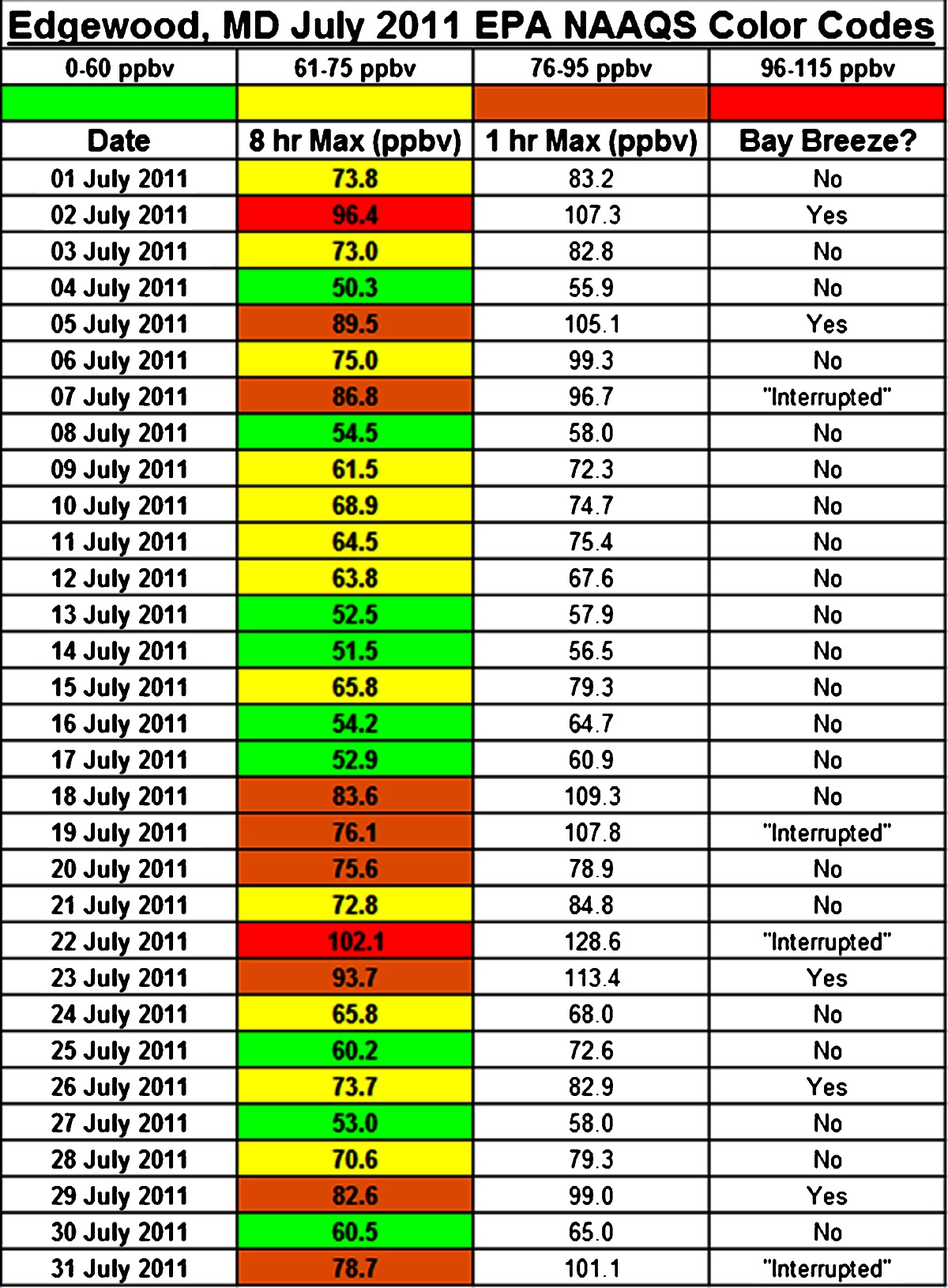
July 2011 8 and 1-h O_3_ maxima by day. 8-h Max boxes are color-coded according to the NAAQS

**Fig. 2:**
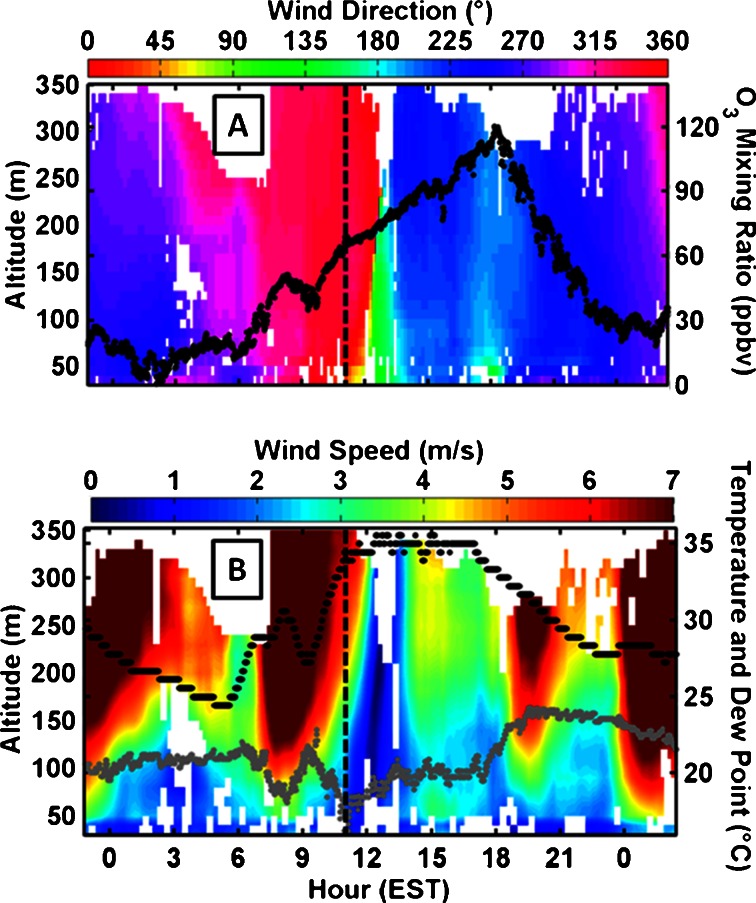
23 July bay breeze effect on wind direction with height (**a**, *colors*), Edgewood surface O_3_ (**a**, *black dots*), wind speed with height (**b**, *colors*), surface temperature (**b**, *black dots*), and dew point (**b**, *grey dots*). Bay breeze frontal passage occurred at approximately 1130 EST (*vertical dashed line*)

An example of the bay breeze’s effect on surface O_3_, wind direction and speed, temperature and dew point is shown for 23 July in Fig. 2. The bay breeze front passed through the Edgewood site at approximately 1130 EST as the wind direction veered to southerly directions. The dew point increased by approximately 4 °C and the temperature plateaued at 34 °C with the frontal passage, a sign of the cooler and moister modified air mass originating over the water being advected over the land. The bay breeze appeared to have moved through Edgewood as a wedge, evidenced by the shift in wind and stagnation first noticed at the surface, then with height as time progresses. This can be expected as temperature gradients closest to the surface are normally greater than those above, and the resulting forcing is also greater closer to the surface. Surface O_3_ increases throughout the day and finally peaks at 1830 EST, leading to an exceedance with an 8 h average of 94 ppbv O_3_. This late day peak in O_3_ (after 1700 EST) occurred on three of five bay breeze days at Edgewood and is notable because photochemical production of O_3_ at these hours should be well beyond its daily potential maximum.

**Fig. 3:**
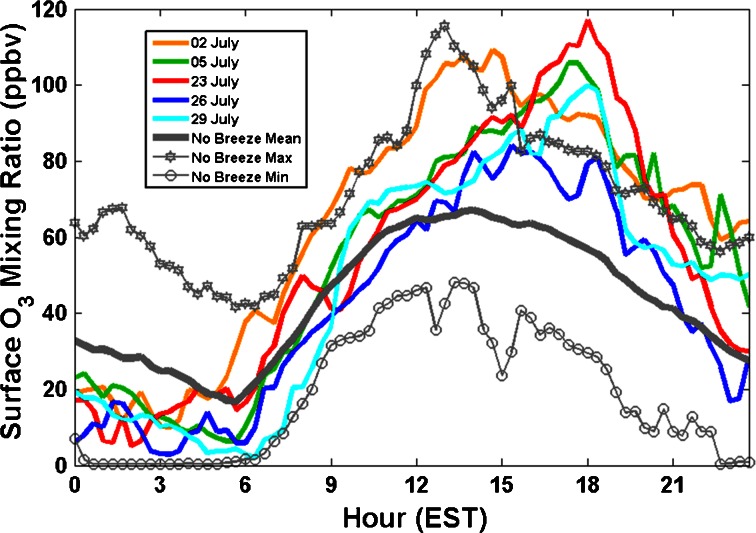
Comparison of surface O_3_ on bay breeze days (*colors*) and non-bay breeze days (*grey*). Twenty minutes averages of O_3_ on each bay breeze day are shown against the minimum, mean, and maximum for every 20 min period of the day for all non-bay breeze days

Figure 3 compares the diurnal cycle of surface O_3_ at Edgewood over the five bay breeze days with the total range diurnal cycle of surface O_3_ for the 22 non-bay breeze days in July 2011. Data were binned into 20-min averages according to time of day. Four of the five bay breeze days were observed to have higher O_3_ than any of the other 22 non-bay breeze days during the period of 1640 to 1900 EST. The maximum 1-h average O_3_ occurred approximately 3 h later for all 5 days as opposed to the 22 non-bay breeze days, which generally peaked in O_3_ around 1300–1400 EST. The change in surface O_3_ for each day from 0800 to 1300 EST and from 1300 to 1800 EST shows that in both time periods, average accumulation rate of O_3_ on bay breeze days (0800–1300 EST: 8.1 ppbv hr^−1^; 1300–1800 EST: 2.4 ppbv hr^−1^) is significantly greater than on non-bay breeze days (0800–1300 EST: 5.0 ppbv hr^−1^; 1300–1800 EST: −2.0 ppbv hr^−1^) with 95 % confidence. All confidence intervals were derived using a bootstrap sampling method with 10,000 iterations (Efron 1979; Efron and Tibshirani 1993).

### Chesapeake Bay measurements

Boundary layer heights (BLHs) from P3-B spirals were determined from four different vertical potential (θ) and virtual potential (θ_v_) temperature gradient methods based on the work by Heffter (1980). The Heffter (1980) method can overestimate boundary layer and mixed layer heights (Berman et al. 1999), given that the θ inversion necessary to calculate these heights must be quite strong. With the possibility of moisture gradients and complex and weaker internal stable layers over the bay, θ_v_ was also considered, which takes into account buoyancy added to an air parcel by water vapor. The inversion criteria were also modified slightly. The different boundary layer heights are estimated by the level closest to the surface having:

1. ∆θ/∆z ≥ 5 K km^−1^ and θ_top_−θ_base_ ≥ 2 K; (Heffter 1980)
2. ∆θ_v_/∆z ≥ 5 K km^−1^ and θ_vtop_−θ_vbase_ ≥ 2 K
3. ∆θ_v_/∆z ≥ 5 K km^−1^ and θ_vtop_−θ_vbase_ ≥ 1 K
4. ∆θ_v_/∆z ≥ 2 K km^−1^ and θ_vtop_−θ_vbase_ ≥ 1 K

where ∆θ/∆z is the potential (1) or virtual potential (2–4) temperature lapse rate and θ_top_ and θ_base_ is the potential or virtual potential temperature at the top and bottom of a layer. Only profiles over the bay that reached a minimum altitude of 400 m were considered. PBL heights were obtained from four to six P3-B spirals depending on the method used (spirals typically started at ~3 km, leading some methods to fail to mark a BLH), over the Chesapeake Bay and compared to coincident half hour averaged MPL-derived PBL heights at Edgewood to examine possible differences in mixing heights over land and water. Methods 1 and 2, both which were able to pick out four boundary layer heights from P3-B spirals, did not statistically differ from the MPL-derived boundary layer heights at Edgewood and had average absolute differences of 22.2 % and 16.8 %, respectively. Methods 3 (five profile BLHs) and 4 (six profile BLHs) were both statistically different from MPL boundary layer heights to 95 % confidence, and were lower by averages of 35.3 % and 58.4 % respectively.

The two weaker θ_v_ criteria, methods 3 and 4, were required to calculate a mixed layer height from a P3-B profile from 26 July, a bay breeze day. A 1 K inversion in θ_v_ from 640 m to 520 m was enough to trap O_3_ near the surface with 43 ppbv measured at 600 m and 73 ppbv measured at 300 m. In addition, a P3-B profile from 11 July over the Chesapeake revealed a decrease in θ_v_ of 0.5 K from 300 m to 250 m, with a corresponding increase in O_3_ of 63 ppbv to 72 ppbv near the surface. None of the four methods for determining boundary layer heights marked this shallow and weak inversion on 11 July. Several weaker stable layers often exist within the first few kilometers that can trap high O_3_ near the bay water surface.

While the heights at which the strong inversion required by Heffter (1980) were marked matched well with the MPL-derived BLHs at Edgewood, the shallower and weaker inversions picked out by methods 3 and 4 can be enough to trap near-surface O_3_ over water and highlight the potential differences between mixing heights at coastal areas and over water surfaces as well as potential issues with MPL BLH determination methods in coastal areas.

**Fig. 4:**
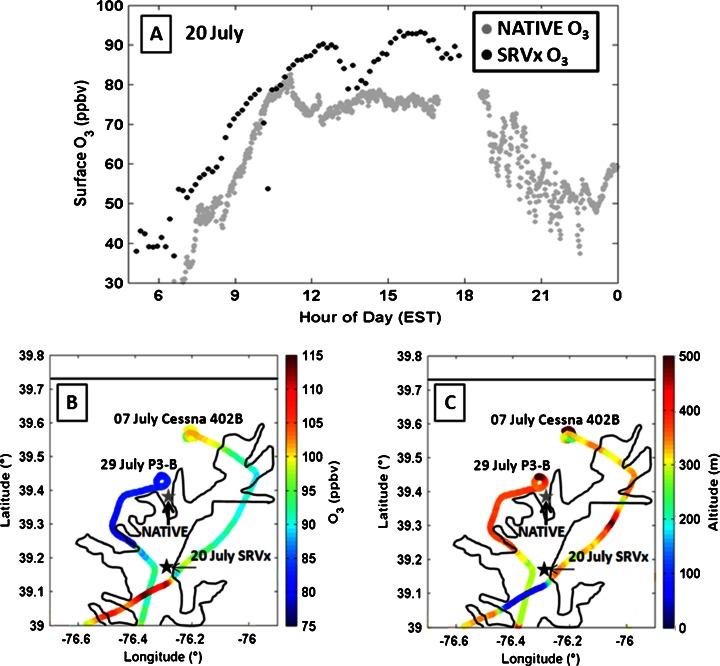
Surface O_3_ on the SRVx (**a**, *black dots*, averaged to 10 min) and at NATIVE (**a**, *grey dots*), O_3_ from the NASA P3-B and UMD Cessna 402B (**b**, *colors*) and flight altitude (**c**, *colors*) for three different days. SRVx surface O_3_ (**a**) was taken on 20 July and flights (**b**, **c**) are 07 July for the UMD Cessna, and 29 July for the NASA P3-B. Downward spikes in SRVx O_3_ measurements are likely O_3_ titration in fresh NO plumes from the ship’s engine exhaust

Figure 4 presents three separate occasions when large land to water O_3_ gradients were observed. The Cessna 402B and P3-B performed transects of the Chesapeake on 07 and 29 July, respectively (Fig. 4b, c). On 07 July, an “interrupted” day, the Cessna observed an O_3_ increase from 88 ppbv to 114 ppbv as it descended from 290 m over land to 60 m over the bay at 1400 EST. On 29 July, a bay breeze day, the P3-B observed an increase in O_3_ from 80 ppbv to 96 ppbv as it descended from 380 m over land to 270 m over the water at 1445 EST.

The SRVx collected measurements in the Chesapeake Bay approximately 25 km south of Edgewood (Fig. 4a; 39.170°, −76.325°) from 0730 to 1700 EST on 20 July. A sharp gradient in O_3_ of 15.4 ppbv averaged from the hours of 1500–1800 EST is observed over this short distance from the Edgewood site to the ship. High surface O_3_ and large vertical gradients were also observed between the SRVx and the Cessna 402B on 20 July. The SRVx and Cessna measured O_3_ mixing ratio gradients of 16 ppbv between the surface and 600 m at 1230 EST, 10 ppbv between the surface and 300 m at 1500 EST, and 17 ppbv from the surface to 300 m at 1645 EST with the higher O_3_ measured by the ship on the water surface. Ozone gradients exist both vertically over the bay and horizontally from the land to the water. Both of these are caused by the aforementioned shallow stable layers formed over water, trapping surface O_3_. The horizontal and vertical gradients in O_3_ and shallow, weak layers of stability over the Chesapeake Bay water surface call for additional soundings and chemical measurements over water in this region in the future.

### Temporal and spatial evolution of bay breeze O_3_

**Fig. 5:**
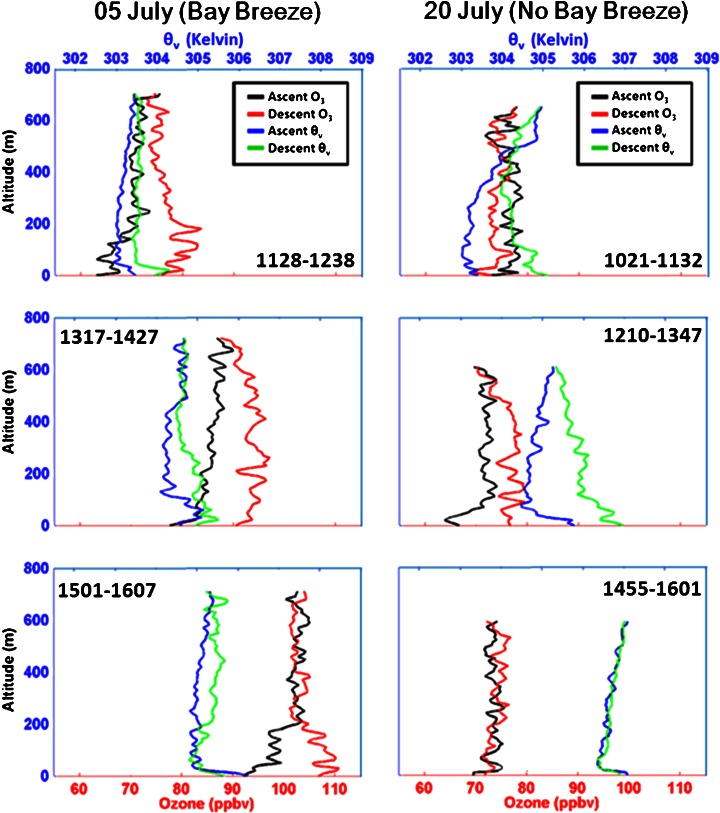
Eagle Point tethered balloon flights on 05 July (bay breeze) and 20 July (no bay breeze) showing progression of O_3_ (ascent: *black*, descent: *red*) and virtual potential temperature (ascent: *blue*, descent: *green*) on two exceedance days. Flight times in EST are labeled on each profile and data are bin-averaged every 10 m. Chesapeake Bay effects are observed with a stable layer containing high O_3_ in the lowest 200 m of the last profile on 05 July

A case-study comparison of 2 days, one with a bay breeze (05 July) and one without (20 July), both of which violated the EPA 8-h standard for O_3_, is shown in Fig. 5. Tethered balloon profiles at Eagle Point illustrate the time-evolution of O_3_ structures and vertical stability as influenced by the Chesapeake Bay. Profiles for each day were taken at comparable times and differences in both stability and O_3_ are observed as a function of time. The bay breeze passage occurred at approximately 1100 EST on 05 July. Ozone increases over time with each profile on bay breeze days such as 05 July with the advection of O_3_-rich air from the bay. Surface O_3_ peaks with highest photochemical production in the early afternoon on non-bay breeze days, then levels or decreases in the evening hours whereas O_3_ mixing ratios are continuously increasing, likely through advection on bay breeze days. The heating of the lowest levels occurs more quickly on 20 July, which can assist vertical mixing and dilution of O_3_, whereas the bay breeze acts to moderate daytime heating on 05 July. Evidence of a TIBL can be seen in the last profile shown for 05 July as a shallow layer of higher stability and higher O_3_ mixing ratios are seen in the lowest 150 m.

**Fig. 6:**
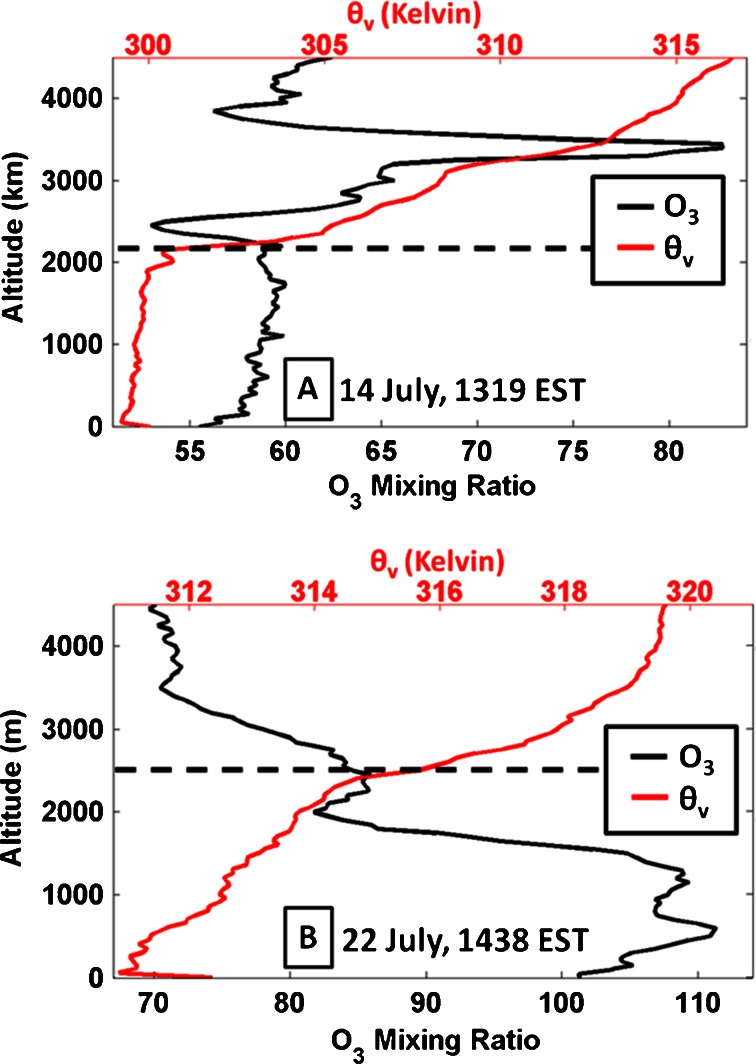
Surface to 4.5 km profiles of O_3_ (*black*) and virtual potential temperature (*red*) from two ozonesonde flights on 14 July (**a**) and 22 July (**b**). 22 July was an “interrupted” day with the bay breeze regaining strength in the afternoon and evening hours after a midday thunderstorm. *Horizontal dashed lines* mark the estimated tops of the boundary layer using the Heffter (1980) method. Launch times are noted on each profile and data are bin-averaged every 50 m

Further effects of complex near-surface layers and a TIBL are found in an ozonesonde sounding following the bay breeze frontal passage at Edgewood on 22 July (Fig. 6). This sounding is compared with a relatively clean and well mixed profile from 14 July. The layers of stability and comparably high O_3_ on 22 July can be seen close to the surface within the low mixing height of the TIBL (marked at 400 m by BLH method 4) as the air mass is advected onshore by the bay breeze. The 14 July vertical profiles are much more uniform with respect to θ_v_ and O_3_, as expected in a well-mixed PBL. This mixing alleviates the build-up of O_3_ at the surface. While the Heffter (1980) BLH method picks a reasonable location for the top of the boundary layer on 14 July (2.1 km), its calculation of BLH on 22 July (2.5 km) shows its shortcomings at times when distinct layers exist. This layering of stability and high O_3_ again gives evidence that the TIBL confounds the vertical distribution of O_3_ on bay breeze days.

**Fig. 7:**
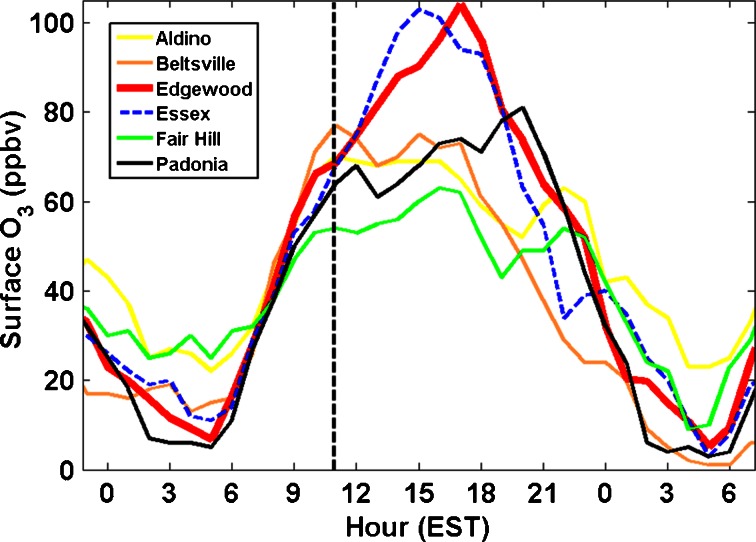
Hourly surface O_3_ at six DISCOVER-AQ stations on 05 July. The initiation of the bay breeze at Edgewood is shown as the *vertical dashed line*, occurring around 1100 EST

**Fig. 8:**
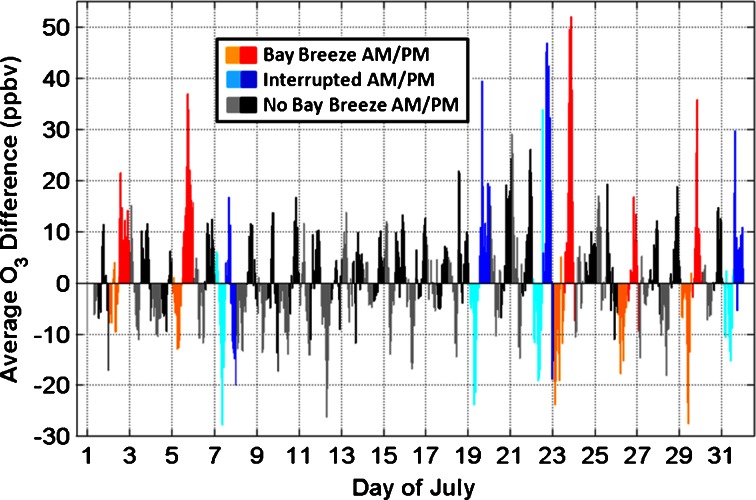
1-h O_3_ difference between Edgewood and the average of five nearby MDE O_3_ sites for bay breeze days (*orange* and *red*), “interrupted” days (*cyan* and *blue*), and non-bay breeze days (*grey* and *black*). AM is defined as 0100–1200 EST and PM is defined as 1300–2400 EST

Surface O_3_ measurements at Edgewood are compared to five nearby MDE stations (Aldino, Beltsville, Essex, Fair Hill, and Padonia) for 05 July in Fig. 7. The bay breeze initiated at Edgewood at 1100 EST and contributed to a violation of the EPA standard at Edgewood and Essex, the latter being another location susceptible to bay breeze effects not fully studied here. Figure 7 highlights the magnitude of the effect a bay breeze can have on surface O_3_ as well as the horizontal variability it induces between the six studied DISCOVER-AQ locations. Figure 8 shows the entire month of average hourly O_3_ differences amongst Edgewood and the five other MDE sites for July 2011. On bay breeze and “interrupted” days, Edgewood consistently displayed hourly O_3_ much higher than the average hourly O_3_ of the other five MDE sites. On non-bay breeze days in Fig. 8 the differences amongst the locations are much less. When considering the 8-h averaged O_3_, these values were statistically the same on non-bay breeze days amongst the six DISCOVER-AQ sites with an average difference of 0.3 ppbv O_3_ between Edgewood and the five other sites. This difference jumps to 10.8 ppbv on bay breeze and “interrupted” days and is statistically different from non-bay breeze days with 95 % confidence. These analyses show how Edgewood consistently observes higher afternoon surface O_3_ than nearby locations during bay breeze events. It has been described how large-scale meteorological conditions favorable for bay breeze initiation are also conducive to surface O_3_ production, but the areas directly affected by the recirculation of chemically aged air in the bay breeze observe O_3_ mixing ratios higher than regional mixing ratios. This localized, elevated O_3_ drives exceedances in this region, and is often the cause of comparably poor air quality at Edgewood.

**Fig. 9:**
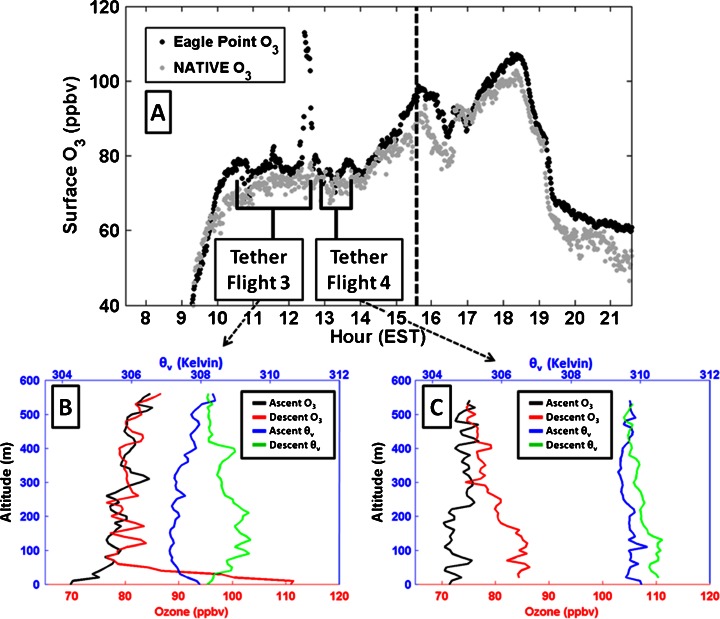
Surface O_3_ at Eagle Point (*black*, **a**) and NATIVE (*grey*, **a**), for 29 July with corresponding Eagle Point tethered balloon flights (**b**, **c**, color scheme same as in Fig. 5) marked by launch start and end. The bay influence is seen in the spike in Eagle Point surface O_3_ as well as the stable surface layer and higher O_3_ in the lowest 100 m at the end of tethered balloon flight 3 (**b**). Bay breeze initiation at NATIVE is marked by the *vertical black dashed line*. Tether profile data are bin-averaged every 10 m

Bay breeze impacts are also apparent on scales much smaller than the inter-site distance. 29 July was identified as a bay breeze day at NATIVE, with the frontal passage characterized by the bay breeze criteria at NATIVE (and Eagle Point) occurring around 1530 EST. Evidence shows that Eagle Point was also impacted by the stable, high O_3_ bay breeze air mass several hours earlier around 1230 EST (Fig. 9). A spike in O_3_ was accompanied by a brief wind shift from northerly to southerly, a 2 °C drop in temperature, and a 3 g kg^−1^ rise in specific humidity in 30 min at the surface at Eagle Point. The very shallow (<100 m) layer in which the effects of the Chesapeake air mass are seen in Fig. 9 demonstrates how O_3_ can be concentrated in the TIBL at the immediate coast (Eagle Point is less than 500 m west of the water). The cool and moist air mass then moved off the coast as the winds shifted back from the north and humidity, O_3_ and θ_v_ lapse rate returned to previous levels, showcasing the competition between the land/water pressure gradient from thermal contrast and weak synoptic scale forcing from north/northwesterly directions. Coincident measurements at the NATIVE site found no such effect as winds remained light from the north/northwest and O_3_ mixing ratios held steady. Later in the afternoon the bay breeze re-formed, moving back inland passing through Eagle Point and NATIVE. Scales on which circulations like this exist are evidently relevant, and motivate the need for high-resolution meteorological and chemical modeling validation from July 2011.

## Conclusions

The bay breeze has a profound impact on surface O_3_ mixing ratios at Edgewood. The bay breeze consistently contributed to exceedances of the 75 ppbv NAAQS value at Edgewood during July 2011. Eight of the 9 days in which a bay breeze or interrupted bay breeze event was observed at Edgewood exceeded this standard, and the only two Code Red days occurred on bay breeze/“interrupted” days. The bay breeze day that did not exceed the EPA standard fell 1 ppbv short at 74 ppbv. The continuous advection of O_3_ late into the evening toward sunset has implications for the risk of extended exposure to the population. Ozone was found to peak an average of 3 h later when the bay breeze was sustained all day than other days in July 2011. The maximization of O_3_ well past the hours of highest incoming solar radiation around solar noon gives evidence that bay breeze transport is a dominant process at Edgewood, more so than at other sites. This phenomenon appears to play a defining role in Edgewood’s poor air quality relative to other O_3_ monitors in the Baltimore NAA.

The observation of land/water horizontal and vertical gradients of O_3_ over the Chesapeake Bay on four separate days during DISCOVER-AQ 2011 point to a need for more consistent monitoring of air quality over the Chesapeake Bay waters, allowing more statistically stringent analyses to determine if the existence of higher O_3_ mixing ratios over the Chesapeake is commonplace during the summer months. Land-water O_3_ gradients and inhibited vertical mixing over cooler water surfaces are key components of the bay breeze/coastal air quality dynamic.

The bay breeze front often acts as a boundary between terrestrial air masses and O_3_-rich air masses existing over the bay. The event at Eagle Point on 29 July is a good example of such a case. Prior to the development of computer resources sufficient to run models on a high enough resolution to capture the scales on which bay breezes occur, derived indices were relied upon to predict the potential for bay or water-body breezes (Biggs and Graves 1962; Laird et al. 2001; Sikora et al. 2010). The complex coastline and warm (relative to large bodies of water) waters of the Chesapeake Bay make predicting these important small-scale circulations difficult and require fine-scale meteorological modeling for resolution of the bay breeze. This work also expressed the importance of vertical stability over the bay and over land during bay breezes, which has a strong effect on the vertical and horizontal distribution of O_3_. Thus, accurately modeling boundary layer heights in this region is also paramount. In-depth investigations into chemical and meteorological modeling results from DISCOVER-AQ 2011 will be pursued in future research.

A long term study into the potential differences in total and tropospheric column O_3_ should be performed to find statistical differences between bay breeze and non-bay breeze days using either satellite overpass data from the Ozone Monitoring Instrument (OMI) on board the Aura satellite or the continuous ground-based measurements provided by the Pandora instrument (Herman et al. 2009; Tzortziou et al. 2012). Analyses of column trace gases of O_3_ and NO_2_ during DISCOVER-AQ in support of the upcoming NASA Geo-CAPE mission will also be the focus of future research.
